# Leptin decreases the expression of low-density lipoprotein receptor via PCSK9 pathway: linking dyslipidemia with obesity

**DOI:** 10.1186/s12967-016-1032-4

**Published:** 2016-09-23

**Authors:** Ying Du, Sha Li, Chuan-Jue Cui, Yan Zhang, Sheng-Hua Yang, Jian-Jun Li

**Affiliations:** State Key Laboratory of Cardiovascular Disease, Division of Dyslipidemia, Fu Wai Hospital, National Center for Cardiovascular Diseases, Chinese Academy of Medical Sciences and Peking Union Medical College, XiCheng District, Beijing, 100037 China

**Keywords:** Leptin, PCSK9, LDLR

## Abstract

**Background:**

Previous studies have suggested that people with obesity showed elevated serum levels of leptin as well as lipid dysfunction and proprotein convertase subtilisin/kexin type 9 (PCSK9) played an important role in the regulation of lipid metabolism recently. The aim of this study was to determine if leptin participated in regulating the uptake of low-density lipoproteins (LDL) in hepatocytes via PCSK9.

**Methods:**

HepG2 cells were treated with human recombinant leptin. The impact of leptin on cellular low density lipoprotein receptor (LDLR) and PCSK9 protein levels was determined by Western blot. Dil-LDL uptake assay was performed to examine the LDLR function. Specific small interfering RNAs (siRNAs) were used to interfere the expressions of target proteins.

**Results:**

The expression of LDLR and LDL uptake could be significantly down-regulated by leptin treatment while the expressions of PCSK9 and hepatocyte nuclear factor 1α (HNF1α) were enhanced in HepG2 cells. Furthermore, inhibition of PCSK9 or HNF1α expression by siRNAs rescued the reduction of LDLR expression and LDL uptake by leptin. We found that leptin activated the p38 mitogen-activated protein kinase (p38MAPK) signaling pathway. Moreover, the changes of the expressions of HNF1α, PCSK9, LDLR, and LDL uptake induced by leptin could be blocked by p38MAPK inhibitor (SB203580). Additionally, leptin attenuated the up-regulation of LDLR caused by atorvastatin in HepG2 cells.

**Conclusions:**

These findings indicated firstly that leptin reduced LDLR levels in hepatocyte via PCSK9 pathway, suggesting that PCSK9 might be a alternative target for dyslipidemia in the obesity.

**Electronic supplementary material:**

The online version of this article (doi:10.1186/s12967-016-1032-4) contains supplementary material, which is available to authorized users.

## Background

Obesity is one of the most serious chronic diseases worldwide represented by an excess of body fat [1]. A number of previous studies indicated that obesity increased risk for atherosclerotic cardiovascular disease (ASCVD) [2, 3]. Dyslipidemia is the earliest risk factor of ASCVD in obesity [4] which includes the elevated serum levels of low density lipoprotein cholesterol (LDL-C) [5]. It has been demonstrated that elevated LDL-C plays a major role in the initiation and development of atherosclerosis in patients with overweight and obesity [5–7]. Unfortunately, the underlying mechanism responsible for elevated LDL-C levels in obesity is not fully understood.

As well established, low density lipoprotein receptors (LDLRs) in the surface of hepatocyte membranes function as critical regulators for circulating LDL-C homeostasis mainly through controlling the rate of liver uptake and the clearance of LDL particles [8, 9]. Several physiological factors which could affect the hepatic LDLR level are recognized. For instance, resistin, which was increased in both circulation and adipose-tissue adipocytes in obesity [10, 11], could reduce LDLR expression in human hepatocytes mediated in part by proprotein convertase subtilisin-kexin type 9 (PCSK9) [12]. In fact, PCSK9 is a secreted protein which plays a key role in regulation of LDL-C level in the circulation by binding directly to the hepatic LDLRs and blocking their recycling via promoting its degradation in lysosome [13]. So far, there are two best-described trans activator of PCSK9 gene expression in hepatocytes, sterol response element binding proteins (SREBP) [14] and hepatocyte nuclear factor 1 alpha (HNF1α) [15].

Besides resistin, leptin is another increased physiological factor in obesity [16] which also comes from adipose tissue [17]. The major function of leptin is that it decreases food intake and increase energy consumption by acting on specific hypothalamic nuclei CART and NPY [18]. Recently, studies in vitro and in vivo have suggested that leptin could also be involved in the pathophysiology of atherosclerosis [19–21]. Furthermore, clinical data have shown the plasma leptin level as an independent predictor for CVD incident [22]. As such, we hypothesized that hyperleptinemia might be another potential mechanism linking obesity to ASCVD and leptin could regulate the expression of LDLR in the liver just like resistin. Leptin has been reported to stimulate p38 mitogen-activated protein kinase (p38MAPK) pathway in different cell types [23–25] and the p38MAPK pathway also participated in the regulation of physiological processes associated with atherosclerosis [25, 26]. Therefore, we supposed that whether p38MAPK pathway could play a role in the regulation of PCSK9 and LDLR by leptin. In addition, statins are the major class of drugs to treatment patients with increased serum LDL-C by upregulating the LDLR expression in hepatocytes [12]. It might be, therefore, interesting to investigate whether leptin could diminish the enhanced LDLR expression induced by atorvastatin treatment. These results will help us to find out whether leptin could affect the statin-induced increase of LDLRs.

In this study, the major goal was to determine if human leptin played a role in regulating the uptake of LDL by PCSK9 in hepG2 cells for the sake of exploring the potential links of leptin with PCSK9 in obesity.

## Methods

### Cell culture and treatment

The human hepatoma cell line HepG2 was purchased from Cell Resource Center, IBMS, CAMS/PUMC. HepG2 cells were cultured in 10 % fetal bovine serum (FBS) (Gibco)-containing DMEM (Gibco) supplemented with 1 % NEAA (Life technologies), 1 % penicillin- streptomycin at 37 °C, 5 % (v/v) CO_2_. HepG2 cells were treated with human recombinant leptin protein (R&D Systems, Minneapolis, MN, USA) at various doses (0, 5, 25, 50, 100 and 200 ng/ml) for 24 h or with 50 ng/ml leptin for various times (0, 6, 12, 24, 48 h). In other experiments, the p38MAPK inhibitor-SB203580, was administered at 10 μM, either alone or combined with 50 ng/ml leptin for 24 h. Atrovastatin (Sigma, MO) was administered at 10 μM, either alone or combined with 50 ng/ml leptin for 24 h.

### Western blots

Cultured cells were collected with cell lysis buffer (Beyotime, Shanghai, China). Protein samples were separated by precast NuPAGE Novex 4–12 % (w/v) Bis–Tris gels (Life technologies, Carlsbad, CA, USA) and then transferred onto nitrocellulose membrane using the iBlotTM dry blotting system as described by the manufacturer (Invitrogen, Carlsbad, CA, USA). Membranes were blocked in TBST buffer (20 mM Tris, pH 7.5, 150 mM NaCl, 0.1 % tween 20) containing 5 % milk for 1 h at room temperature and incubated with primary antibodies specific for LDLR (Biovision, Mountain View, CA), PCSK9 (Cayman Chemicals, MI), HNF1α (Cell Signaling) and GAPDH (Abcam) overnight at 4 °C, and then incubated with a secondary antibody conjugated with horseradish peroxidase (HRP) for 2 h at room temperature. Blots were developed using chemoluminescence (ECL, Thermo Fisher Scientific, Waltham, MA, USA) on FluorChem M image system.

### DiI-LDL uptake assay

HepG2 cells were changed to serum-free media and incubated with 10 μg/ml DiI-LDL (lifetech) for 24 h at 37 °C in the dark. After incubation, the cells were washed with PBS and fixed in the presence of a 4 % paraformaldehyde, and the nuclei were subsequently stained with Hoechst dye. Finally, the cells were examined with fluorescence microcopy (DMI-4000B, Leica).

### Small interfering ribonucleic acid (siRNA) transfection

PCSK9 and HNF1α Stealth siRNA duplexes are followings: PCSK9 Stealth siRNA duplexes (Life tech, Carlsbad, CA, USA) targeting sequences: 5′-GAC AUC AUU GGU GCC UCC AGC GAC U-3′ and 5′-AGU CGC UGG AGG CAC CAA UGA UGU C-3′ and HNF1α Stealth siRNA duplexes (Life tech, Carlsbad, CA, USA) targeting sequences: 5′-UCG AUA CCA CUG GCC UCA ATT-3′ and 5′-UUG AGG CCA GUG GUA UCG ATT-3′. The negative controls siRNA Duplexes (Life tech, Carlsbad, CA, USA) were used as a control. siRNA were transfected into HepG2 cells using Lipofectamine TM RNAiMAX (Life tech, Carlsbad, CA, USA) according to manufacturer’s protocol.

### Statistical analysis

Results were presented as mean ± SEM at least three independent experiments. Significant differences between control and treatment groups were assessed by One-way ANOVA with proper posttest or Student two-tailed *t* test.

## Results

### Leptin down-regulates expression of LDLR and up-regulates expression of PCSK9

**Fig. 1:**
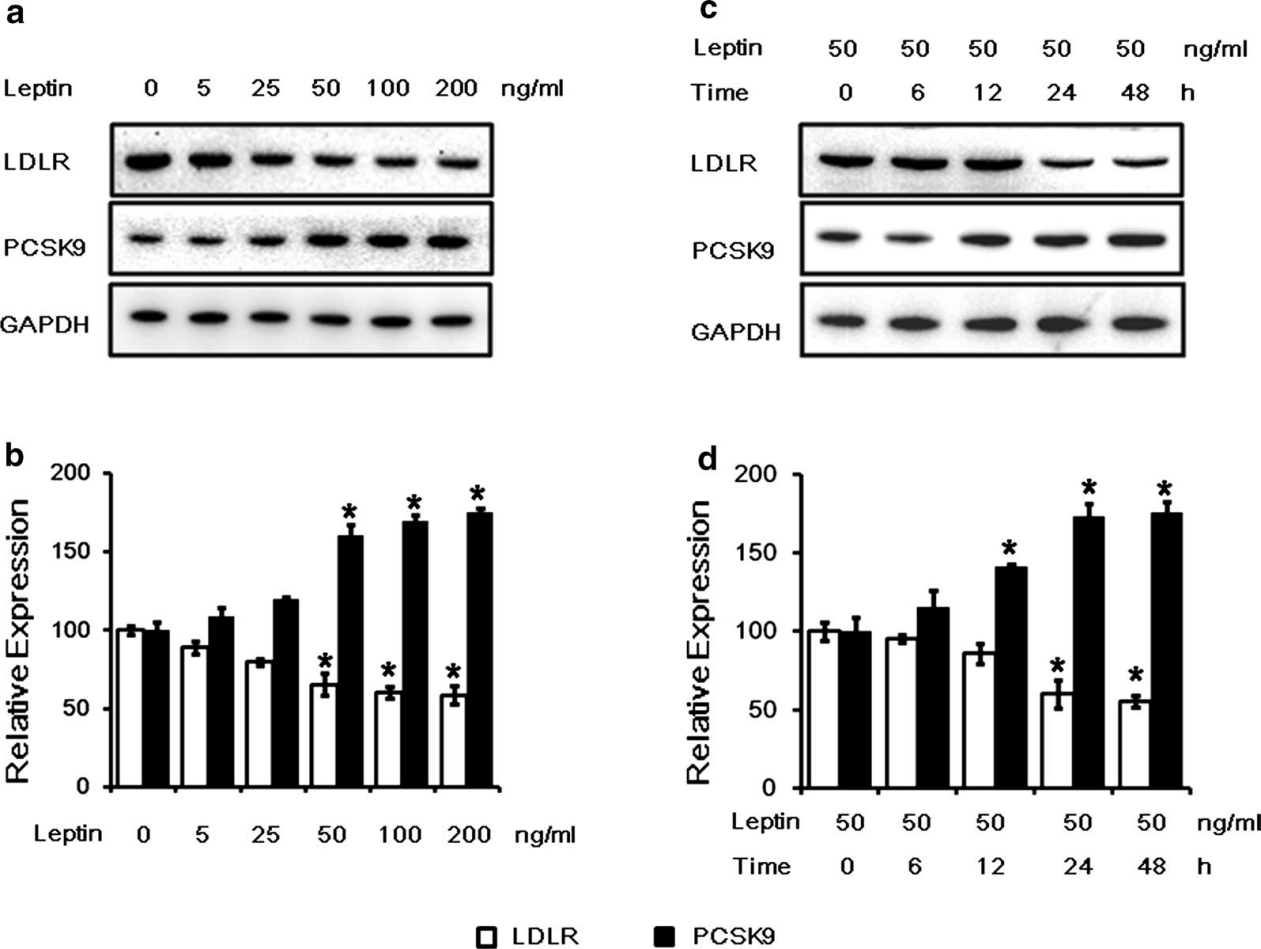
The effects of leptin on LDLR and PCSK9 protein levels in HepG2 cells. **a** *Western blot* analysis of leptin on LDLR and PCSK9 protein levels in HepG2 cells treated with leptin (0, 5, 25, 50, 100 and ng/mL) for 24 h. **b**The normalized intensities of LDLR and PCSK9 versus GAPDH are presented as the mean ± SD of three independent dose-dependent experiments. **c** *Western blot* analysis of leptin (50 ng/ml) on LDLR and PCSK9 protein levels in HepG2 cells treated with leptin for 0, 6, 12, 24, 48 h. **d** The normalized intensities of LDLR and PCSK9 versus GAPDH are presented as the mean ± SD of three independent time-dependent experiments. *p < 0.05 represent significant differences compared to the vehicle-treated cells

To investigate the effect of leptin on the expression of LDLR and PCSK9, we treated HepG2 cells with different concentrations of leptin (0, 5, 25, 50, 100 and 200 ng/ml) for 24 h. As a result, the LDLR protein levels were decreased and the PCSK9 protein levels were increased by leptin stimulation in a dose-dependent manner (Fig. 1a, b). Significant changes in LDLR and PCSK9 protein expression, compared with vehicle-treated cells, were observed at 50, 100, 200 ng/ml of leptin treatments. Subsequently, we used 50 ng/ml of leptin to stimulate HepG2 cells with different times (0, 6, 12, 24, 48 h). The impact of leptin on LDLR and PCSK9 appeared in a time-dependent manner (Fig. 1c, d). The results showed that the PCSK9 expression was significantly enhanced by leptin treatment after 12 h and the LDLR expression was significantly reduced after 24 h.

### PCSK9 inhibition blocks the effect of leptin on LDLR expression and LDL uptake

**Fig. 2:**
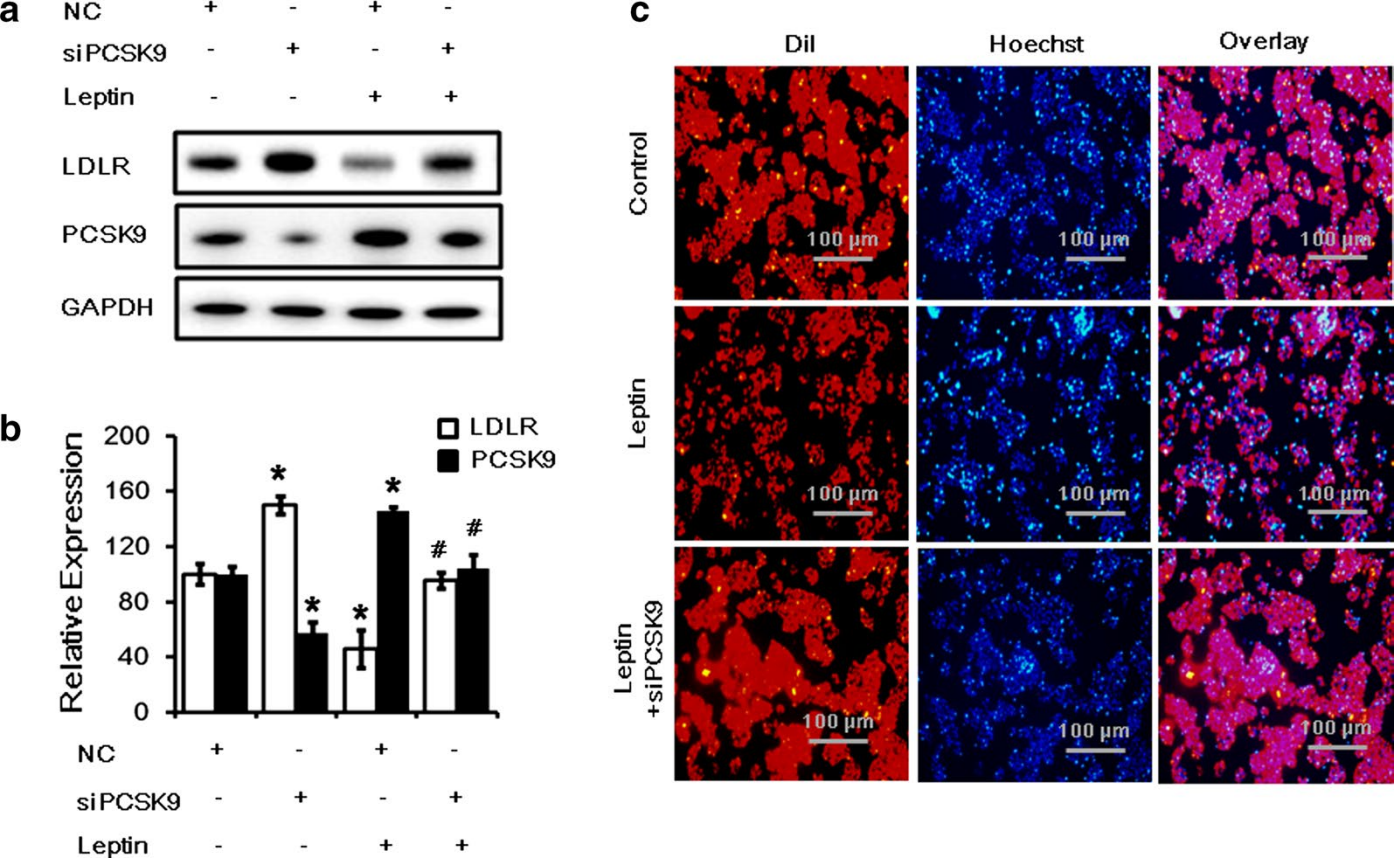
Inhibition of PCSK9 expression returned LDLR expression and LDL uptake during leptin treatment in HepG2 cells. **a** *Western blot* analysis of LDLR and PCSK9 protein levels in HepG2 cells transfected with siPCSK9 (40 nM) and then treated with leptin (50 ng/mL) for 24 h. **b**The normalized intensities of LDLR and PCSK9 versus GAPDH are presented as the mean ± SD of three independent experiments. **c** Representative fluorescence microscopy images of cell-associated Dil-LDL (*red*), Hoechst-stained nuclei (*blue*), and the overlay. *p < *0.05* represent significant differences compared to the vehicle-treated cells. ^#^ *p* < *0.05* represent significant differences compared to the leptin-treated cells

We further investigated whether the down-regulation of LDLR by leptin was mediated by PCSK9. HepG2 cells were pre-treated with with negative control siRNAs (40 nM), which did not target any gene, or the PCSK9 siRNA for 24 h before the treatment of leptin. Our results showed that inhibition of endogenous PCSK9 expression using the PCSK9 siRNA (40 nM) abrogated the suppression of the LDLR expression (Fig. 2a, b) and LDL uptake (Fig. 2c) by leptin after 24 h treatment.

### HNF1α inhibition blocks the effect of leptin on LDLR expression and LDL uptake

**Fig. 3:**
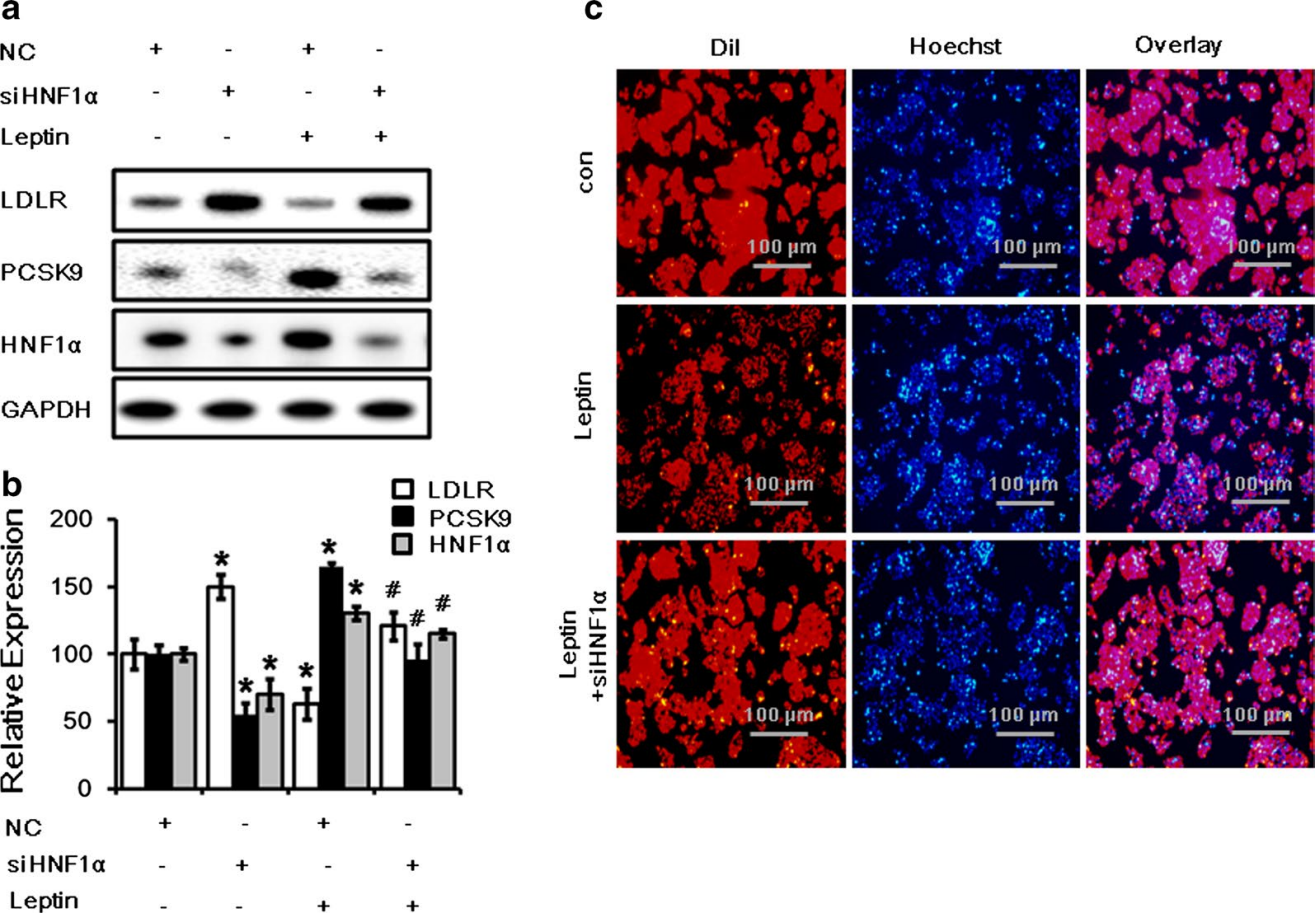
Inhibition of HNF1α expression returned LDLR and PCSK9 expression and LDL uptake during leptin treatment in HepG2 cells. **a** *Western blot* analysis of LDLR, PCSK9 and HNF1α protein levels in HepG2 cells transfected with siHNF1α (40 nM) and then treated with leptin (50 ng/mL) for 24 h. **b** The normalized intensities of LDLR, PCSK9 and HNF1α versus GAPDH are presented as the mean ± SD of three independent experiments. **c** Representative fluorescence microscopy images of cell-associated Dil-LDL (*red*), Hoechst-stained nuclei (*blue*), and the overlay. *p < *0.05* represent significant differences compared to the vehicle-treated cells. ^#^ *p* < *0.05* represent significant differences compared to the leptin-treated cells

HNF1α is an upstream regulator of PCSK9 which can bind to the promoter of PCSK9 and directly regulate PCSK9 expression [15]. To further confirm that leptin promotes PCSK9 expression by the activation of HNF1α, we transfected HepG2 cells with HNF1α siRNA for 24 h before leptin treatment. The data suggested that inhibition of endogenous HNF1α expression by the HNF1α siRNA (40 nM) could also abrogate the decrease in LDLR expression and increase in PCSK9 expression (Fig. 3a, b) as well as the suppression of LDL uptake (Fig. 3c) by leptin after 24 h treatment.

### Leptin down-regulates LDLR and up-regulates PCSK9 through p38MAPK pathway

**Fig. 4:**
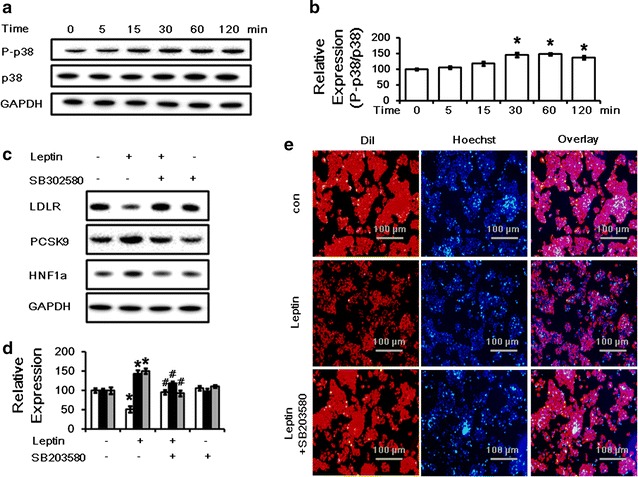
Leptin modulates LDLR and PCSK9 protein levels and LDL uptake through a p38MAPK-dependent pathway. **a** *Western blot* analysis of phosphorylated-p38 and total p38 protein levels in HepG2 cells treated with 50 ng/ml leptin for 0, 5, 15, 30, 60, 120 min. **b** The normalized intensities of phosphorylated-p38 versus total p38 are presented as the mean ± SD of three independent experiments. **c** *Western blot* analysis of LDLR, PCSK9 and HNF1α protein levels in HepG2 cells treated with leptin (50 ng/mL) alone or combined with SB 203580 (10 μM) for 24 h. **d** The normalized intensities of LDLR, PCSK9 and HNF1α versus GAPDH are presented as the mean ± SD of three independent experiments. **e** Representative fluorescence microscopy images of cell-associated Dil-LDL (*red*), Hoechst-stained nuclei (*blue*), and the overlay. *p < *0.05* represent significant differences compared to the vehicle-treated cells. ^#^ *p* < *0.05* represent significant differences compared to the leptin-treated cells

The MAPK signaling cascades have been shown to be induced by leptin treatment in different cells [23–25]. In this study, we found that phosphorylation of p38 could be induced by leptin in HepG2 cells in a time-dependent manner (Fig. 4a, b). To investigate whether p38MAPK pathway was involved in the regulation of LDLR by leptin, HepG2 cells were co-cultured with the p38MAPK specific inhibitor-SB203580 (10 μM) alone or combined with leptin. The data indicated that inhibition of the p38MAPK pathway abolished the decrease in LDLR expression and the increase in PCSK9 and HNF1α expression (Fig. 4c, d) as well as the suppression of LDL uptake (Fig. 4e) by leptin after 24 h treatment.

### Leptin inhibits the up-regulation of LDLR mediated by atorvastatin

**Fig. 5:**
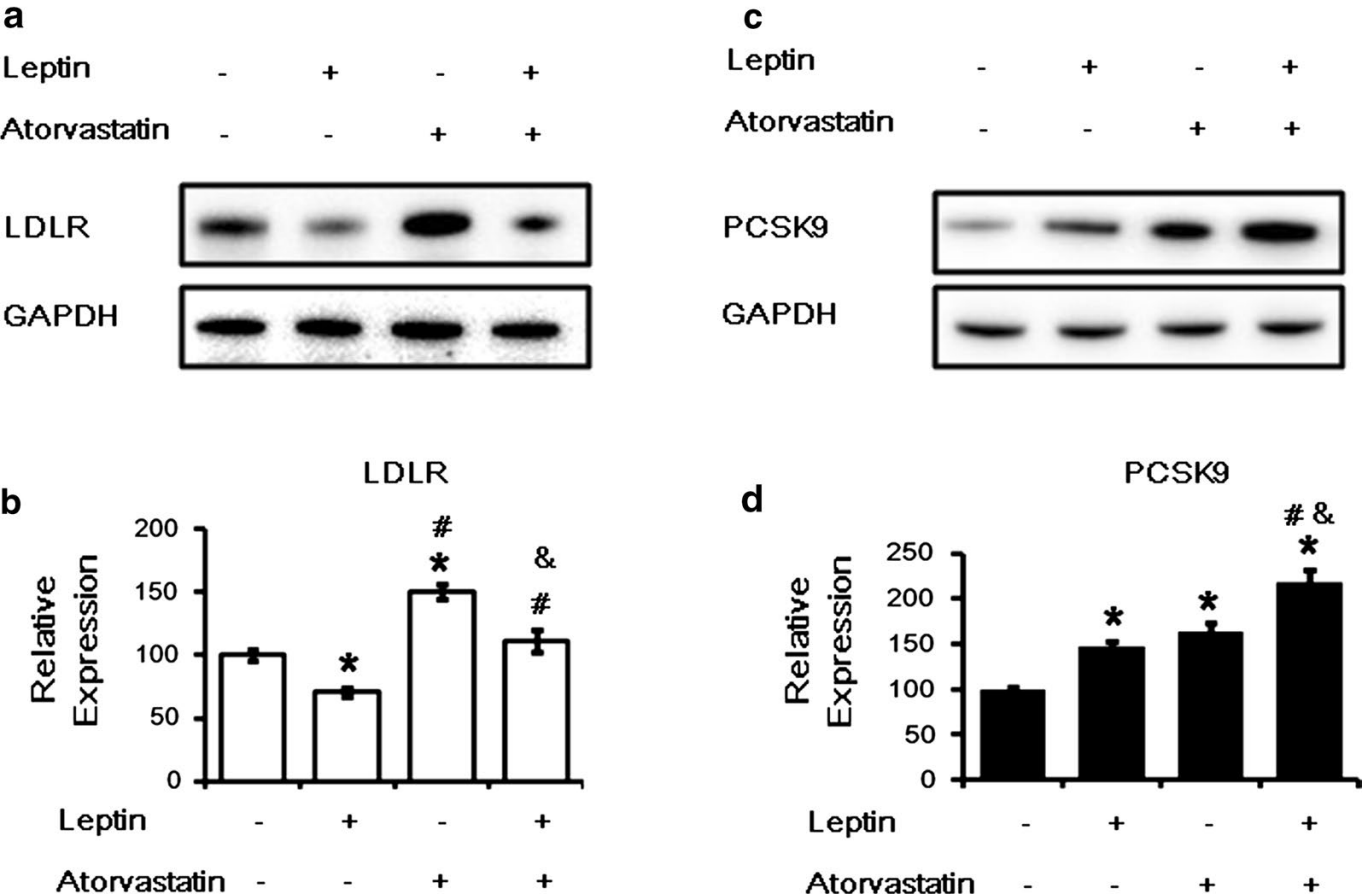
The effect of a combination of leptin and atrovastatin in HepG2 cells. **a** *Western blot* analysis of LDLR protein levels in HepG2 cells treated with leptin (50 ng/mL) alone or combined with atorastatin (10 μM) for 24 h. **b** The normalized intensity of LDLR versus GAPDH are presented as the mean ± SD of three independent experiments. **c** *Western blot* analysis of PCSK9 protein levels in HepG2 cells treated with leptin (50 ng/ml) alone or combined with atorastatin (10 μM) for 24 h. **d** The normalized intensity of PCSK9 versus GAPDH are presented as the mean ± SD of three independent experiments. *p < *0.05* represent significant differences compared to the vehicle-treated cells. ^#^ *p* < *0.05* represent significant differences compared to the leptin-treated cells. ^&^ *p* < *0.05* represent significant differences compared to the atorvastatin-treated cells

Additionally, we tested whether leptin inhibited the normal statin-mediated up-regulation of hepatic LDLR levels. HepG2 cells were treated with atrovastatin (10 μM) alone or combined with leptin (50 ng/ml) for 24 h. Consistent with previous studies [12], our results showed that leptin diminished the increase in hepatic LDLR expression induced by atorvastatin treatment (Fig. 5a, b) and increase in cellular PCSK9 protein compared with atorvastatin treatment alone (Fig. 5c, d).

## Discussion

In this study, we, for the first time, demonstrated that leptin significantly increased PCSK9 expression and suppressed LDLR in HepG2 cells. The major novel findings of the present study were as follows: (1) leptin could suppress LDLR level, LDL uptake and elevate PCSK9 expression; (2) the changes of LDLR, PCSK9 levels and LDL uptake were regulated by enhanced expression in HNF1α and the elevated activation of p38MAPK pathway; (3) leptin could abolish the statin-induced up-regulation of hepatic LDLR level. This study revealed a novel role of leptin in the regulation of hepatic LDLR and PCSK9 expression, which might provide additional information to understand the mechanism responsible for dyslipidemia in obesity.

It is well known that leptin is an adipose tissue-derived hormone, which plasma level is positive correlated with the body fat mass [16, 17]. The major function of leptin is to regulate human body weight and energy homeostasis by the brain [18]. Considering the roles of leptin in physiological processes associated with cardiovascular disease, such as blood pressure [27], platelet aggregation [28], arterial thrombosis [29], angiogenesis [30], and inflammatory vascular responses [31], it, logically, may have a close relationship with the development of CVD [32]. Interestingly, study from Kwakernaak et al. found that plasma leptin had a positive association with plasma PCSK9 in healthy people [33]. Their results suggested that leptin might be involved in the regulation of hepatic PCSK9 expression. Therefore, illuminating the relationship of leptin and PCSK9 in vitro is the major goal of this study.

In the beginning of this study, we found that leptin could cause the decrease of LDLR expression and LDL uptake (Additional file 1: Figure S1) in HepG2 cells. Then, we sought to illuminate the mechanisms by which leptin regulates the hepatic LDLR levels. Definitely, PCSK9 have been reported to regulate LDL levels by mediating LDLR protein degradation [13]. That is, PCSK9 could bind to the extracellular domain of the LDLRs and then lead LDLRs to lysosome-mediated degradation instead of recycling to the cell surface [13]. Therefore, PCSK9 stood a good chance to mediate the regulation of hepatic LDLR levels. Consisting with our assumption, the present study showed that PCSK9 levels were increased by leptin stimulation. Subsequently, we further investigated the role of PCSK9 in the leptin-mediated reduction in LDLR levels. In our study, inhibition of PCSK9 gene expression were carried out by transfecting PCSK9 siRNA into HepG2 cells. Negative control siRNAs were used as controls, which did not affect expression PCSK9 and LDLR (Additional file 2: Figure S2). And the relative transfection efficiency was shown by using the positive control–GAPDH siRNA and it was great in our experiments (Additional file 3: Figure S3). As a result, we found that the suppression of LDLR expression and LDL uptake by leptin stimulation could be reversed by the inhibition of PCSK9 expression. These findings suggested that leptin could suppress the LDLR expression by increasing hepatic PCSK9 level.

HNF1α is one of the transcriptional regulation factors which regulates PCSK9 expression by binding to its promoter [15]. To determine whether HNF1α was involved in the regulation of LDLR and PCSK9 by leptin, HNF1α siRNAs were used to knockdown its expression. It was found that elevation of PCSK9 expression and the reduction of LDLR expression and function by leptin stimulation could be abolished by the inhibition of HNF1α expression. It is well known that another transcription factor, SREBP2 is the most important trans-activator of PCSK9 which can also induce LDLR expression [14]. Hence, we also examined the SREBP2 expression under leptin treatment for 24 h. Interestingly, we found that there was no significant change of SREBP2 expression by leptin in both mRNA and protein level (Additional file 4: Figure S4 and Additional file 5: Figure S5). Although the exact reason for this unique phenomenon is unclear, we supposed that SREBP2 may not be involved in the regulation of LDLR mediated by PCSK9 under leptin treatment.

Leptin has been reported to signal via various kinases. The activity of p38MAPK pathway is also modulated by leptin in different cell types. For instance, leptin reduced the activity of the Na+/K+ ATPase significantly by activating p38MAPK in Caco-2 cells [23]; leptin could inhibit glucose intestinal absorption by activation of p38MAPK [24]; Besides, leptin also could activate human B cells to secrete cytokines via activation of p38MAPK/ERK1/2 signaling pathways, which may contribute to its inflammatory and immunoregulatory properties [34]. Furthermore, the p38MAPK pathway has also been demonstrated to be participated in the regulation of physiological process associated with atherosclerosis. Activation of p38MAPK pathway could regulate interleukin-16 -induced migration and invasion of vascular smooth muscle cells [26] and be involved in the suppression in oxidized low-density lipoprotein- induced endothelial cell apoptosis by paenonl [25]. Therefore, we examined whether p38MAPK pathway could be activated by leptin treatment. Interestingly, we found that the phosphorylation of p38 could be increased by leptin (Fig. 4a, b). In addition, the inhibition of p38MAPK pathway by its inhibitor SB203580 could abolish the elevation in PCSK9 expression and the suppression of LDLR expression and LDL uptake by leptin stimulation (Fig. 4c, d, e; Additional file 6: Figure S6). On the contrary to our results, Pham DD et al. recently reported that nerve growth factor (NGF) could increase LDLR expression by stimulation of p38MAPK and activation of caspase-3 and SREBP2 cleavage in hepatocytes [35]. In this study, the activation of SREBP2 was mediated by signaling pathway triggered by ligand-activated p75NTR which were induced by p38MAPK and caspase-3. In their study, Caspase-3 activation was caused by p38MAPK following NGF stimulations. In our study, leptin did not change the levels of SREBP2 proteins including both uncleaved and mature form (Additional file 5: Figure S5) but activate the p38MAPK. The possible reason is that leptin could suppress caspase-3 activity in HepG2 cells [36]. Thereby the p75NTR could not be activated and then SREBP2 cleavage would not be affected by letpin.

Additionally, statins are the major-class therapy currently used for patients with increased serum LDL-C by upregulating the LDLR expression in hepatocytes [12]. Therefore, it might be interesting to investigate whether leptin could diminish the enhanced LDLR expression induced by atorvastatin treatment. Our results showed that leptin could block the increase of LDLR expression induced by atorvastatin treatment (Fig. 5). This inhibition in the statin-induced LDLR up-regulation by leptin was partially contributed by increasing PCSK9 protein level. These results suggested that inhibitors for leptin might enhance hepatic LDLR expression and reduce plasma LDL-C level when it was administered combined with statins. Further studies are needed to conform our findings and test our hypothesis in the future.

## Conclusion

**Fig. 6:**
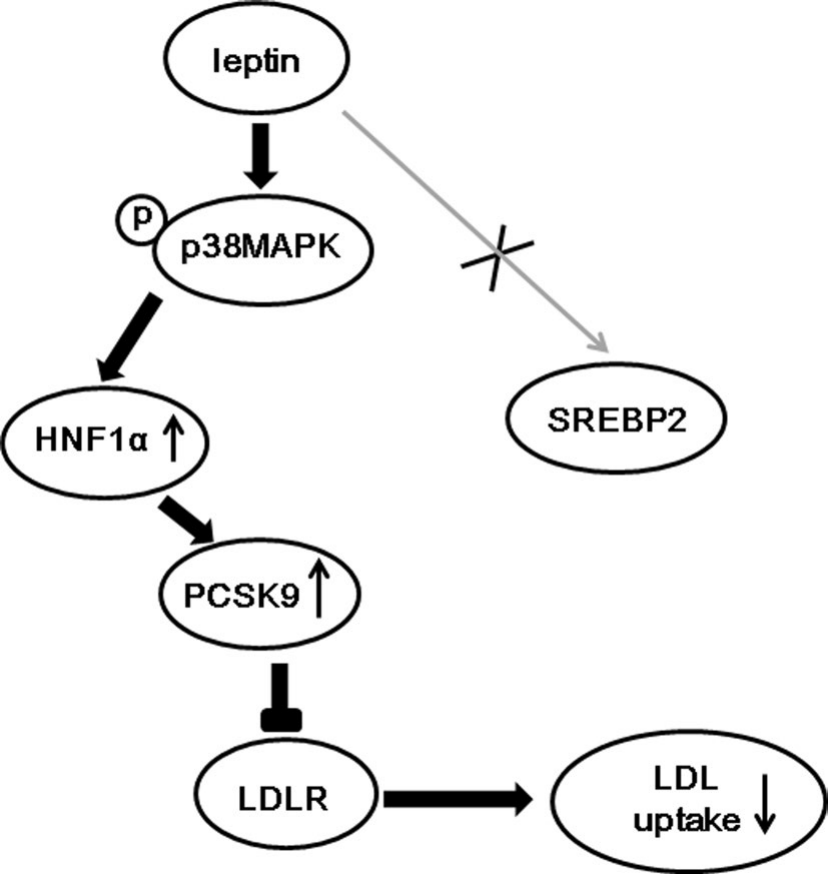
Summary of pathways by leptin on PCSK9 and LDLR expression in HepG2 cells. Stimulation of p38MAPK pathway by leptin increased expression of nuclear HNF1α which could bind to PCSK9 promoter, leading to increase PCSK9 expression in HepG2 cells. The increased PCSK9 leaded to elevated LDLR degradation and inhibited LDL uptake

In conclusion, leptin could down-regulate the expression and function of LDLR and up-regulate the expression of PCSK9 through the p38MAPK- and HNF1α-dependent mechanism (Fig. 6). Our findings supported that the regulation of LDLR and PCSK9 by leptin might be potential mechanism linking obesity to ASCVD.

## Additional files

10.1186/s12967-016-1032-4 Time course of LDL uptake response to leptin treatment in HepG2 cells. Representative fluorescence microscopy images of cell-associated Dil-LDL (red), Hoechst-stained nuclei (blue), and the overlay in HepG2 cells treated with leptin (50 ng/ml) for 0, 6, 12, 24, 48 h.
10.1186/s12967-016-1032-4 Effect of negative control siRNA on LDLR and PCSK9 expression. Real time PCR analysis of LDLR and PCSK9 mRNA levels in HepG2 cells transfected with negative control siRNA for 24 h. Con, control (vehicle); NC, negative control.
10.1186/s12967-016-1032-4 Knockdown efficiency of GAPDH siRNA in HepG2 cells. Real time PCR analysis of GAPDH mRNA levels in HepG2 cells transfected with GAPDH siRNA for 24 h. **p* < 0.05 represent significant differences compared to the vehicle-treated cells.
10.1186/s12967-016-1032-4 Examination of the effect of leptin on PCSK9, LDLR, HNF1α and SREBP2 mRNA Levels in dose- or time-dependent manner in HepG2 cells. (A) Real time PCR analysis of LDLR, PCSK9, HNF1α and SREBP2 mRNA levels in HepG2 cells treated with leptin (0, 5, 25, 50, 100 and ng/mL) for 24 h. (B) Real time analysis of LDLR, PCSK9, HNF1α and SREBP2 mRNA levels in HepG2 cells treated with leptin (50 ng/ml) for 0, 6, 12, 24, 48 h. **p* < 0.05 represent significant differences expression compared to the vehicle-treated cells.
10.1186/s12967-016-1032-4 Effect of leptin on expression of SREBP2. (A) Western blot analysis of SREBP2 protein levels in HepG2 cells treated with leptin (50 ng/mL) for 24 h. (B) The normalized intensities of uncleaved and mature SREBP2 versus GAPDH are presented as the mean ± SD of three independent experiments.
10.1186/s12967-016-1032-4 Effect of p38MAPK inhibitor with different concentrations on LDLR and PCSK9 expression. (A) Western blot analysis of LDLR and PCSK9 protein levels in HepG2 cells treated with leptin (50 ng/mL) alone with SB203580 (1 μM or 10 μM) for 24 h. (B) The normalized intensities of LDLR and PCSK9 versus GAPDH are presented as the mean ± SD of three independent experiments. **p* < 0.05 represent significant differences compared to the vehicle- treated cells. ^#^ *p* < 0.05 represent significant differences compared to the leptin-treated cells.

## Abbreviations

ASCVD: atherosclerotic cardiovascular disease
HepG2: human hepatocarcinoma cell line 2
HNF1α: hepatocyte nuclear factor 1 alpha
LDL: low density lipoprotein
LDLR: low density lipoprotein receptor
MAPK: mitogen-activated protein kinase
PCSK9: proprotein convertase subtilisin-kexin type 9
SREBP: sterol response element binding proteins
