# Supplementary figures and images for: Leptin decreases the expression of low-density lipoprotein receptor via PCSK9 pathway: linking dyslipidemia with obesity
Source: J Transl Med. 2016 Sep 23;14:276. doi: 10.1186/s12967-016-1032-4 (PMC5035475; doi:10.1186/s12967-016-1032-4)

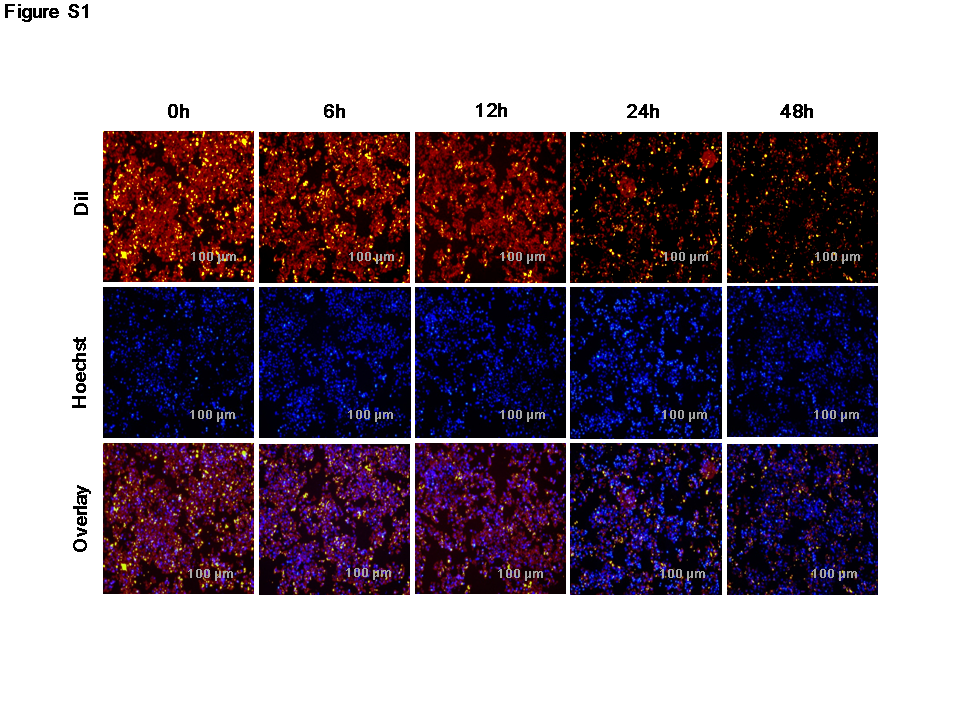

Supplement: Supplementary file 1 — 10.1186/s12967-016-1032-4 Time course of LDL uptake response to leptin treatment in HepG2 cells. Representative fluorescence microscopy images of cell-associated Dil-LDL (red), Hoechst-stained nuclei (blue), and the overlay in HepG2 cells treated with leptin (50 ng/ml) for 0, 6, 12, 24, 48 h. [file 12967_2016_1032_MOESM1_ESM.tif]

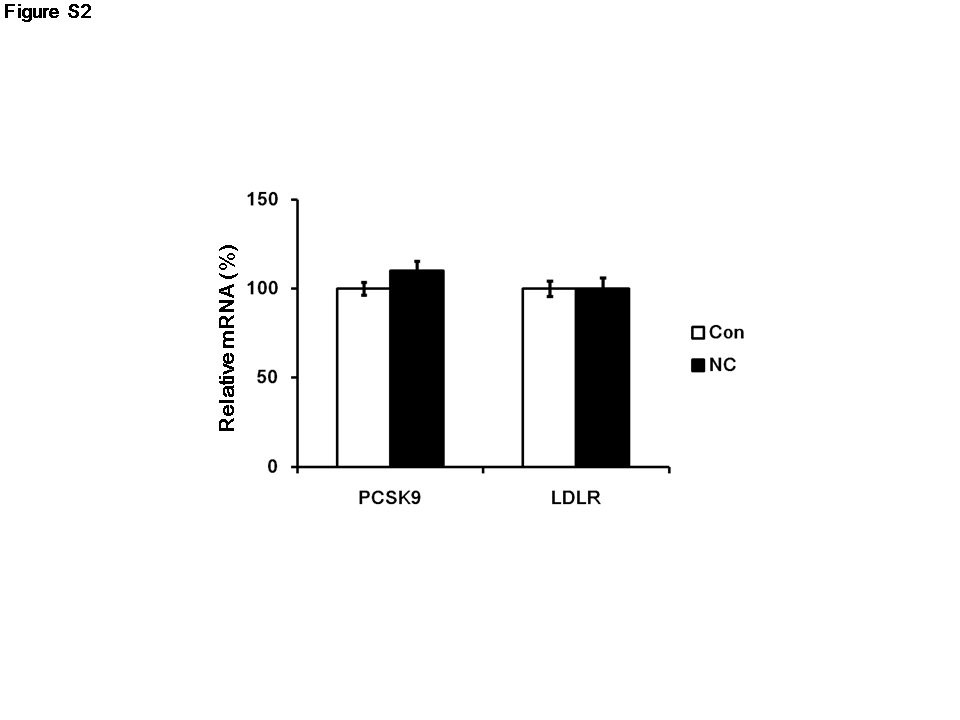

Supplement: Supplementary file 2 — 10.1186/s12967-016-1032-4 Effect of negative control siRNA on LDLR and PCSK9 expression. Real time PCR analysis of LDLR and PCSK9 mRNA levels in HepG2 cells transfected with negative control siRNA for 24 h. Con, control (vehicle); NC, negative control. [file 12967_2016_1032_MOESM2_ESM.tif]

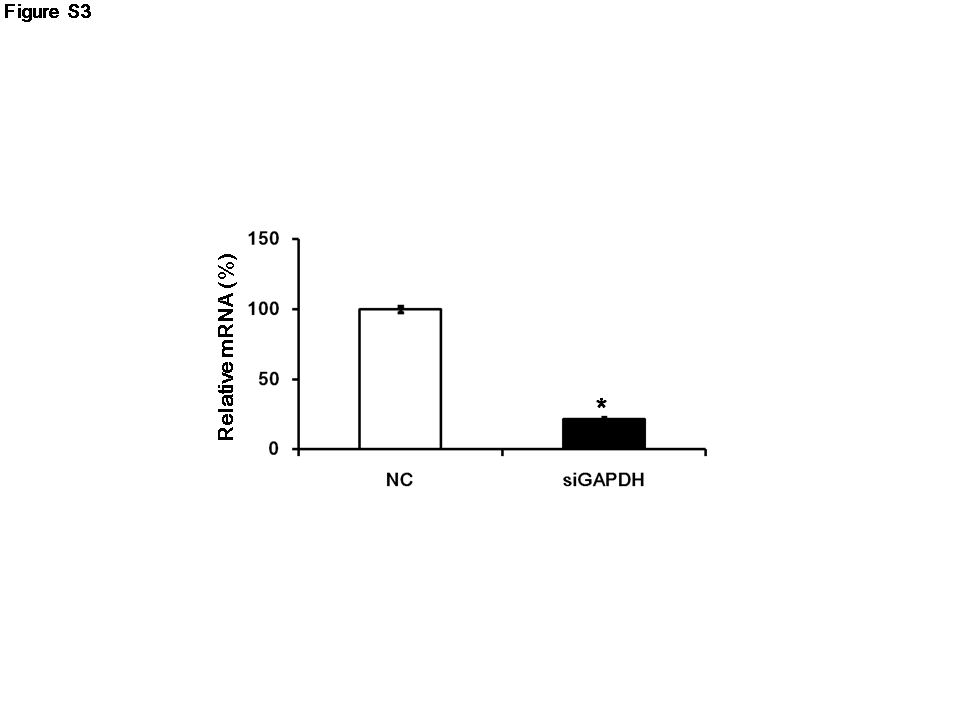

Supplement: Supplementary file 3 — 10.1186/s12967-016-1032-4 Knockdown efficiency of GAPDH siRNA in HepG2 cells. Real time PCR analysis of GAPDH mRNA levels in HepG2 cells transfected with GAPDH siRNA for 24 h. *p < 0.05 represent significant differences compared to the vehicle-treated cells. [file 12967_2016_1032_MOESM3_ESM.tif]

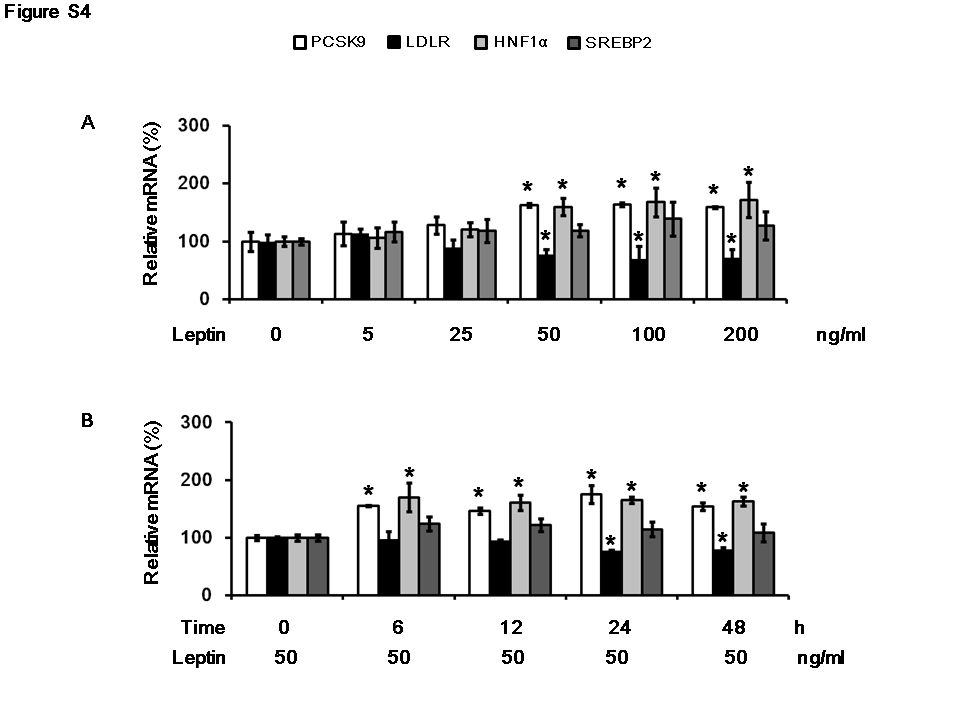

Supplement: Supplementary file 4 — 10.1186/s12967-016-1032-4 Examination of the effect of leptin on PCSK9, LDLR, HNF1α and SREBP2 mRNA Levels in dose- or time-dependent manner in HepG2 cells. (A) Real time PCR analysis of LDLR, PCSK9, HNF1α and SREBP2 mRNA levels in HepG2 cells treated with leptin (0, 5, 25, 50, 100 and ng/mL) for 24 h. (B) Real time analysis of LDLR, PCSK9, HNF1α and SREBP2 mRNA levels in HepG2 cells treated with leptin (50 ng/ml) for 0, 6, 12, 24, 48 h. *p < 0.05 represent significant differences expression compared to the vehicle-treated cells. [file 12967_2016_1032_MOESM4_ESM.tif]

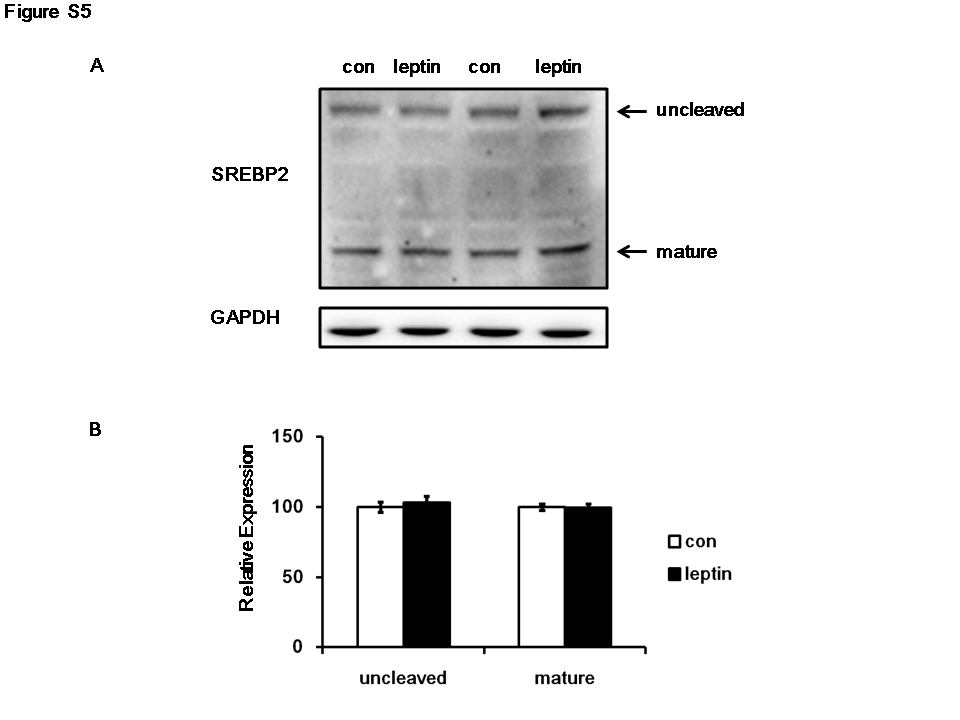

Supplement: Supplementary file 5 — 10.1186/s12967-016-1032-4 Effect of leptin on expression of SREBP2. (A) Western blot analysis of SREBP2 protein levels in HepG2 cells treated with leptin (50 ng/mL) for 24 h. (B) The normalized intensities of uncleaved and mature SREBP2 versus GAPDH are presented as the mean ± SD of three independent experiments. [file 12967_2016_1032_MOESM5_ESM.tif]

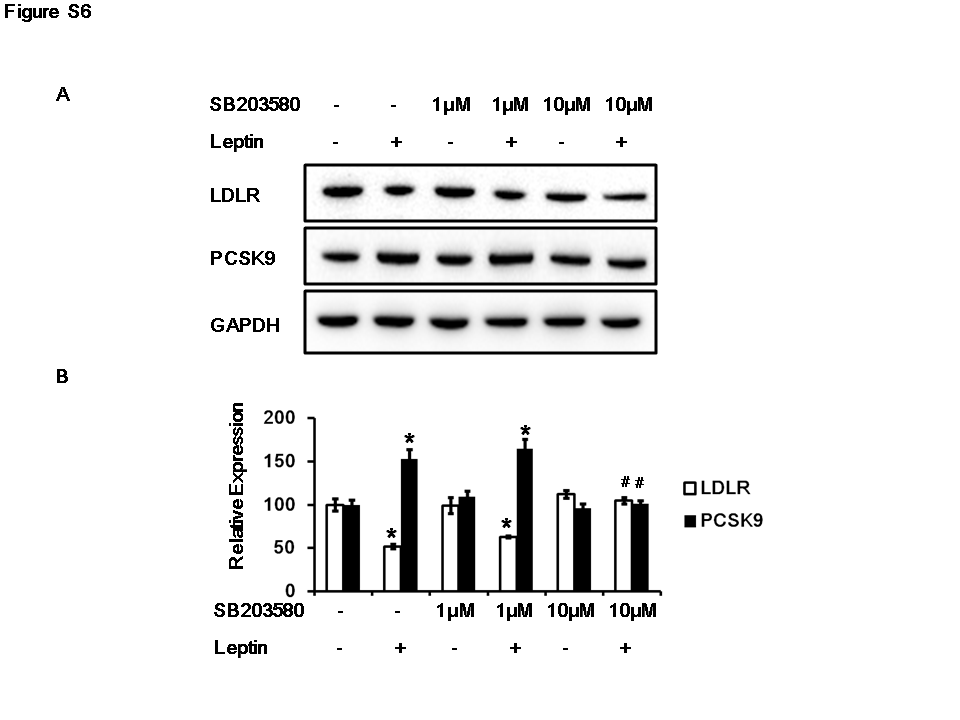

Supplement: Supplementary file 6 — 10.1186/s12967-016-1032-4 Effect of p38MAPK inhibitor with different concentrations on LDLR and PCSK9 expression. (A) Western blot analysis of LDLR and PCSK9 protein levels in HepG2 cells treated with leptin (50 ng/mL) alone with SB203580 (1 μM or 10 μM) for 24 h. (B) The normalized intensities of LDLR and PCSK9 versus GAPDH are presented as the mean ± SD of three independent experiments. *p < 0.05 represent significant differences compared to the vehicle- treated cells. # p < 0.05 represent significant differences compared to the leptin-treated cells. [file 12967_2016_1032_MOESM6_ESM.tif]
